# Neuroprotective Effects of Testosterone in the Hypothalamus of an Animal Model of Metabolic Syndrome

**DOI:** 10.3390/ijms22041589

**Published:** 2021-02-04

**Authors:** Erica Sarchielli, Paolo Comeglio, Sandra Filippi, Ilaria Cellai, Giulia Guarnieri, Alessandra Marzoppi, Sarah Cipriani, Linda Vignozzi, Annamaria Morelli, Mario Maggi

**Affiliations:** 1 Section of Human Anatomy and Histology, Department of Experimental and Clinical Medicine, University of Florence, 50134 Florence, Italy; erica.sarchielli@unifi.it (E.S.); giulia.guarnieri@unifi.it (G.G.); 2 Andrology, Women’s Endocrinology and Gender Incongruence Unit, Department of Experimental Clinical and Biomedical Sciences “Mario Serio”, University of Florence, 50134 Florence, Italy; paolo.comeglio@unifi.it (P.C.); ilaria.cellai@unifi.it (I.C.); alessandra.marzoppi@stud.unifi.it (A.M.); sarahcipriani88@gmail.com (S.C.); linda.vignozzi@unifi.it (L.V.); 3 Interdepartmental Laboratory of Functional and Cellular Pharmacology of Reproduction, Department of Neurosciences, Psychology, Drug Research and Child Health (NEUROFARBA), University of Florence, 50134 Florence, Italy; sandra.filippi@unifi.it; 4 I.N.B.B. (Istituto Nazionale Biostrutture e Biosistemi), 00136 Rome, Italy; mario.maggi@unifi.it; 5 Endocrinology Unit, Department of Experimental Clinical and Biomedical Sciences “Mario Serio”, University of Florence, 50134 Florence, Italy

**Keywords:** inflammation, testosterone treatment, hypothalamus, metabolic syndrome, hypogonadotropic hypogonadism

## Abstract

Metabolic syndrome (MetS) is known to be associated to inflammation and alteration in the hypothalamus, a brain region implicated in the control of several physiological functions, including energy homeostasis and reproduction. Previous studies demonstrated the beneficial effects of testosterone treatment (TTh) in counteracting some MetS symptoms in both animal models and clinical studies. This study investigated the effect of TTh (30 mg/kg/week for 12 weeks) on the hypothalamus in a high-fat diet (HFD)-induced animal model of MetS, utilizing quantitative RT-PCR and immunohistochemical analyses. The animal model recapitulates the human MetS features, including low testosterone/gonadotropin plasma levels. TTh significantly improved MetS-induced hypertension, visceral adipose tissue accumulation, and glucose homeostasis derangements. Within hypothalamus, TTh significantly counteracted HFD-induced inflammation, as detected in terms of expression of inflammatory markers and microglial activation. Moreover, TTh remarkably reverted the HFD-associated alterations in the expression of important regulators of energy status and reproduction, such as the melanocortin and the GnRH-controlling network. Our results suggest that TTh may exert neuroprotective effects on the HFD-related hypothalamic alterations, with positive outcomes on the circuits implicated in the control of energy metabolism and reproductive tasks, thus supporting a possible role of TTh in the clinical management of MetS.

## 1. Introduction

The hypothalamus is a brain region crucially implicated in the control of several physiological processes, including energy metabolism and reproductive function. Although considered to be immune-privileged because of the presence of the blood–brain barrier (BBB), recent findings have shown that the brain is able to sense peripheral metabolic and proinflammatory cues, particularly at the hypothalamic level [1]. In fact, given its anatomical position close to the third ventricle and circumventricular organs, where the BBB is partially interrupted, the hypothalamus is extremely sensitive to circulating factors. This condition makes the hypothalamic neurons susceptible to and reactive to homeostatic modifications, including those related to a persistent systemic inflammatory status, as is the case with obesity and other metabolic derangements [1,2].

High-fat diet (HFD)-induced hypothalamic inflammation—i.e., upregulation of the inflammatory cytokines, including tumor necrosis factor α (TNF-α), interleukin (IL)-1β, and IL-6—was described as early as 2005 in rats fed an HFD for 16 weeks [3] and then confirmed in several animal models [4,5,6,7]. In humans, hypothalamic gliosis and microglial activation were detected by MRI and histological analyses in obese subjects [4,8] and obesity-associated hypothalamic alterations with presence of inflammatory markers were demonstrated using diffusion tensor imaging [9].

An increased hypothalamic inflammation was initially observed in association with HFD and obesity; however, soon after, it was demonstrated that this alteration could be associated with other systemic disorders, even independently of obesity, such as insulin resistance and hypertension. In fact, an increased hypothalamic inflammation was observed following altered peripheral insulin sensitivity and glucose tolerance and blood pressure dysregulation, through an increased renin–angiotensin system activity [10]. Thus, these observations led to postulate an association between hypothalamic inflammation and a more complex clustering of pathological conditions, such as in the construct of metabolic syndrome (MetS). Indeed, MetS is a cluster of metabolic and cardiovascular disorders, including glucose intolerance, visceral obesity, dyslipidemia, and hypertension, leading to an increased risk of diabetes and major adverse cardiovascular events [10].

It is known that obesity and MetS are closely associated with a low-grade, systemic, and chronic inflammatory condition that differs from other causes of chronic inflammation such as autoimmunity [11]. MetS-associated low-grade inflammation is, in fact, present in several tissues, including white adipose tissue [12,13], liver [14,15], skeletal muscle [16,17], and pancreas [18,19]. Very interestingly, some studies on HFD-fed animal models have demonstrated that hypothalamic inflammation appears long before inflammatory events in peripheral tissues and precedes significant body weight gain [20]. Indeed, in rodents, a single day of HFD led to an increased hypothalamic expression of IL-6 and TNF-α and microglial cell activation [21]. Moreover, three days of exposure to HFD raised neuroinflammation, gliosis, and markers of neuronal injury [4]. On the other hand, HFD-induced hypothalamic inflammation is often persistent, and, after returning to a low-fat diet, the recovery of function is slow [22].

Chronic inflammation leads to apoptosis of hypothalamic neurons and, consequently, reduces synaptic inputs in the lateral hypothalamus (LH) and medio-basal hypothalamus (MBH), particularly in the arcuate nucleus (ARC), where neurons of the melanocortin system are located [23]. This system is the principal hub in the control of nutritional status and of energy balance, and it is composed by two neuronal populations with antagonistic functions that reciprocally regulate one another to form a complex neuronal circuit; one subset of neurons expresses the orexigenic neuropeptides agouti-related peptide (AgRP) and neuropeptide Y (NPY), whereas the second subset expresses the anorexigenic peptides proopiomelanocortin (POMC) and cocaine- and amphetamine-regulated transcript (CART). The ARC-located melanocortin neurons are recognized as the main targets of inflammation-induced apoptosis, resulting in an imbalance of the hypothalamic control of body energy homeostasis [2,4].

It is well known that androgens exert inhibitory action on immune cell activity with anti-inflammatory properties and suppressive effects on macrophages, neutrophils, natural killer cells, and T cells [24]. Moreover, some studies showed that testosterone (T) has immune-downregulating properties, associated with an improvement of insulin and leptin sensitivity and other parameters of obesity and MetS [11,25]. We previously demonstrated in a nongenomic animal model of MetS, obtained by feeding male rabbits an HFD, the occurrence of hypothalamic inflammation [7]. This animal model develops all the features of human MetS, including visceral obesity, hypertension, dyslipidemia, glucose intolerance, secondary hypogonadism, i.e., low testosterone (T) and low gonadotropins (LH and FSH), and hepatic alterations as in non-alcoholic fatty liver disease (NAFLD) and non-alcoholic steatohepatitis (NASH) [15,26]. Of note, T decline resulted dose-dependently related to MetS severity, since T decreased as a function of the number of MetS components [27]. Interestingly, T treatment (TTh) in this animal model determined an improvement of several MetS-related alterations [24] and exerted a relevant anti-inflammatory effect in several peripheral organs [15,28,29,30,31].

Given the relevant role played by hypothalamic inflammation as an early player in obesity and MetS-related dysfunctions, and considering that an overt condition of hypogonadotropic hypogonadism is often associated with MetS, this study was aimed at investigating possible neuroprotective actions of T at the hypothalamic level in MetS rabbits.

## 2. Results

### 2.1. MetS Phenotype Induced by HFD and Effects of TTh

HFD rabbits showed a significant increase in all MetS components. In fact, we observed hyperglycemia, glucose intolerance, dyslipidemia, hypertension, and accumulation of visceral fat upon HFD chronic exposure (Table 1). Secondary hypogonadism, characterized by low T and LH, was also present in HFD rabbits (Table 1). These observations were associated with a lower weight of the androgen-dependent gland prostate (Table 1).

TTh (30 mg/kg/week intramuscular for 12 weeks) exerted beneficial effects on mean arterial blood pressure (MAP) and visceral adipose tissue accumulation, as well as on glucose homeostasis, with a significant decrease in fasting glucose levels (Table 1) and a trend to an improved glucose tolerance (oral glucose tolerance test, OGTT, *p* = 0.06 vs. HFD; Table 1). On the contrary, TTh did not affect HFD-induced dyslipidemia (i.e., total cholesterol and triglycerides) and, as expected, LH levels remained suppressed (Table 1). Prostate and seminal vesicle weight was significantly increased by TTh (Table 1). No significant differences were observed in body weight between the three groups (Table 1).

In addition, MetS (more than three MetS components) was present in more than two-thirds of HFD-treated rabbits (70.6%, *p* < 0.001 vs. RD), while TTh significantly decreased this proportion to less than one in five (18.7%, *p* < 0.01 vs. HFD). None of the regular diet (RD)-fed rabbits satisfied MetS criteria.

### 2.2. HFD-Induced Hypothalamic Inflammation and Effects of TTh

**Figure 1:**
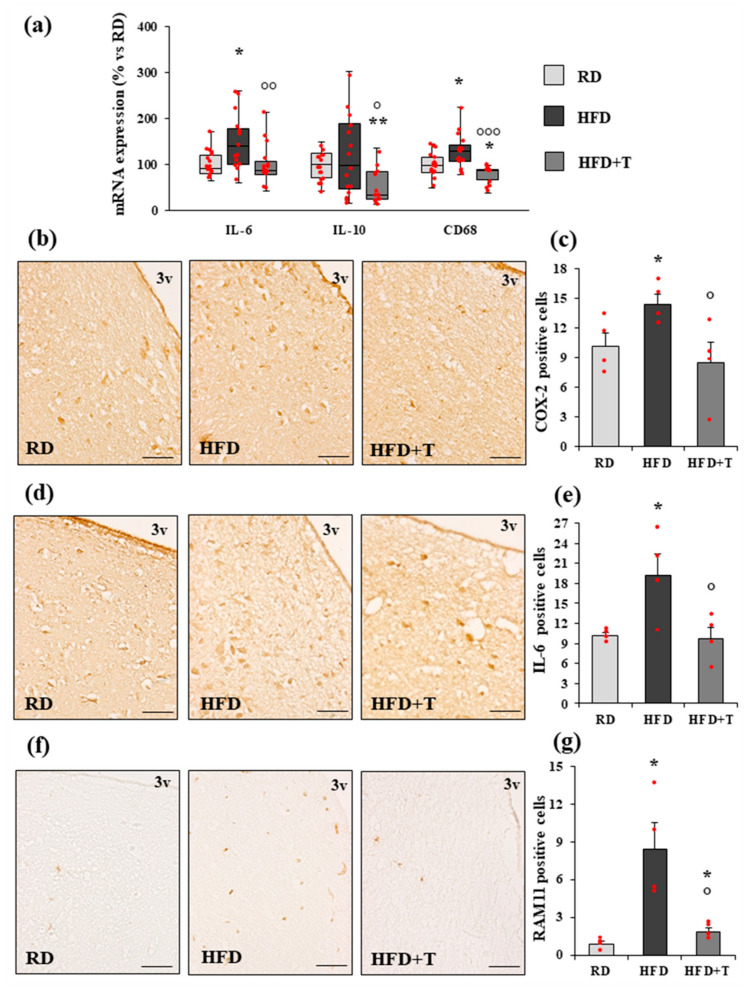
Real-time RT-PCR and immunohistochemical analysis of inflammation markers in rabbit hypothalamus. (**a**) Messenger RNA (mRNA) expression of interleukin IL-6, IL-10, and CD68 genes in RD, HFD, and HFD + T rabbit hypothalamic samples. Data were calculated using the 2^−ΔΔCt^ comparative method, with the 18S ribosomal RNA subunit used as a housekeeping gene for normalization, and they are reported as a percentage vs. RD as the median ± interquartile range (*n* = 16 for RD, *n* = 15 for HFD, *n* = 14 for HFD + T). Statistical analysis between groups was performed with Kruskal–Wallis and post hoc Mann–Whitney nonparametric tests. (**b**–**g**) Representative images of cyclooxygenase-2 (COX-2) (**b**), IL-6 (**d**), and macrophage-specific RAM11 (**f**) staining of coronal hypothalamic sections, including the region lining the third ventricle (3v) (scale bar = 50 μm). The bar graphs show the quantification of COX-2 (**c**), IL-6 (**e**), and RAM11 (**g**) positive cells obtained by counting 10 fields in four different samples from each group (mean ± standard error of the mean (SEM), *n* = 4 for each group). * *p* < 0.05, ** *p* < 0.01 vs. RD; ° *p* < 0.05, °° *p* < 0.01, °°° *p* < 0.001 vs. HFD.

Hypothalamic samples from RD, HFD, and HFD+T rabbits were analyzed by qRT-PCR for messenger RNA (mRNA) expression of genes involved in inflammation and immune response (IL-6, IL-10, CD68). As shown in Figure 1, HFD significantly increased mRNA expression of IL-6 and CD68 genes. TTh strongly counteracted the proinflammatory effect of HFD by decreasing the mRNA expression of all the inflammation-related genes analyzed (Figure 1, panel a).

These findings were also confirmed at the protein level by immunohistochemical analysis with cyclooxygenase-2 (COX-2), IL-6, and macrophage-specific RAM11 antibodies in coronal sections of the anterior and tuberal hypothalamic regions, where neurons controlling energy metabolism are located (Figure 1, panels b–g). As compared to RD, HFD significantly increased the number of COX-2 and IL-6 immunopositive cells in the region lining the third ventricle (Figure 1, panels b–c and d–e, respectively). Accordingly, HFD hypothalamic sections were clearly immunopositive for the macrophage marker RAM11, which was scarcely expressed in sections from RD rabbits (Figure 1, panels f–g). TTh was able to significantly reduce the number of immunopositive cells for the three inflammatory markers analyzed (Figure 1).

**Figure 2:**
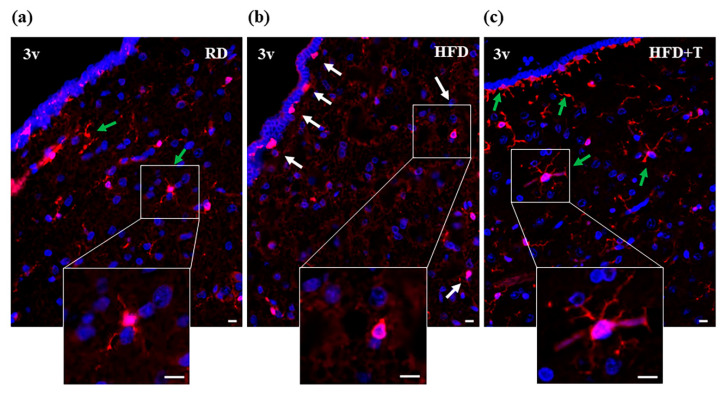
Immunohistochemical analysis of microglia morphology of rabbit hypothalamus. Representative images of hypothalamic coronal sections of the region lining the third ventricle (3v) from RD (**a**), HFD (**b**), and HFD + T (**c**) rabbits, immunostained with an antibody against the microglial marker IBA1 (scale bar = 10 μm). Green arrows indicate cells with ramified morphology, and white arrows indicate cells with activated-amoeboid morphology, as better shown in the zoomed image (scale bar = 10 μm). DAPI (4′,6-diamidino-2-phenylindole)-counterstained nuclei.

To further evaluate the presence of an inflammatory response induced by HFD at the hypothalamic level, we next analyzed the cellular morphology of microglial cells, closely related to their functional status, by staining cells with IBA1 antibody. In the absence of inflammation, microglial cells exhibited a ramified morphology, as observed in RD hypothalamic sections (Figure 2, panel a). On the contrary, when activated by inflammation, microglial cells assumed an amoeboid morphology, as observed in HFD samples (Figure 2, panel b). In hypothalamic sections from TTh, HFD microglial cells no longer exhibited the activated-amoeboid morphology, showing a small cell body and ramified processes similar to RD sections (Figure 2, panel c).

### 2.3. Gene Expression Analysis of Estrogen Receptors, Glucose Metabolism Regulators, and GnRH-Related Markers

**Figure 3:**
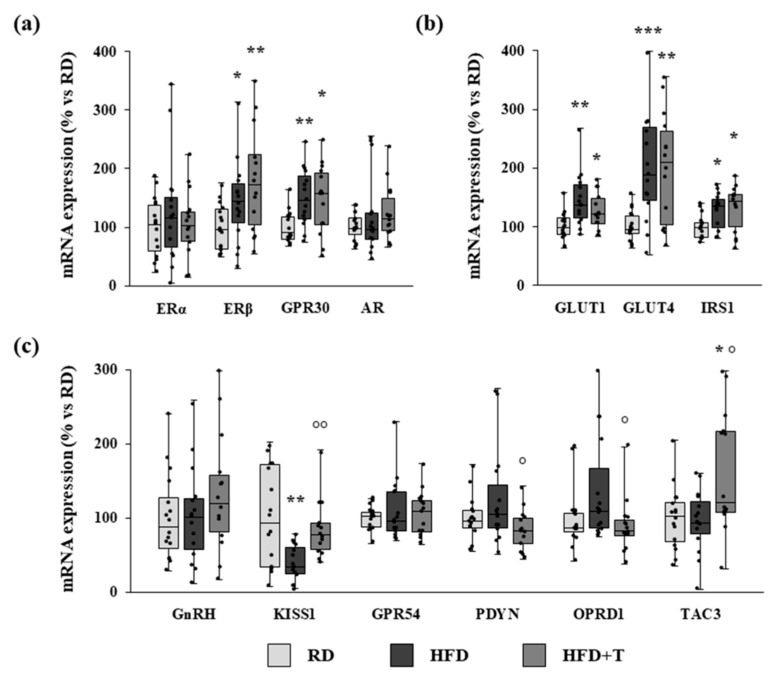
mRNA expression of sex hormone receptors and markers of glucose metabolism and of GnRH neuron function in RD, HFD, and HFD + T hypothalamic samples, as evaluated by qRT-PCR. Panels show analysis of estrogen receptor alpha (ERα), ERβ, GPR30, and androgen receptor (AR) genes (**a**), GLUT1, GLUT4, and IRS-1 genes (**b**) and GnRH, kisspeptin 1 (KISS1), GPR54, dynorphin (PDYN), opioid receptor delta 1 (OPRD1), and tachykinin 3 (TAC3) genes (**c**). Data were calculated using the 2^−ΔΔCt^ comparative method, with the 18S ribosomal RNA subunit used as a housekeeping gene for normalization, and they are reported as a percentage vs. RD as median ± interquartile range (*n* = 16 for RD, *n* = 15 for HFD, *n* = 14 for HFD + T). Statistical analysis between groups was performed with Kruskal–Wallis and post hoc Mann–Whitney nonparametric tests (* *p* < 0.05, ** *p* < 0.01, *** *p* < 0.001 vs. RD; ° *p* < 0.05, °° *p* < 0.01 vs. HFD).

The qRT-PCR analysis showed that the mRNA expression of the estrogen receptors ERβ and GPR30 was significantly increased by HFD (Figure 3, panel a). This increase was not affected by TTh (Figure 3, panel a). ERα and androgen receptor (AR) gene expression was not modified by any treatment (Figure 3, panel a).

An increased expression of genes related to glucose transport and insulin activity (GLUT1, GLUT4, and IRS-1) was also observed in HFD hypothalamic samples and not changed by TTh (Figure 3, panel b).

The expression of a panel of genes known to regulate the GnRH neuron function was also analyzed. Gene expression of KISS1, encoding kisspeptin—one of the main physiological regulators of GnRH secreting neurons—was significantly reduced by HFD, while no significant differences were observed for GnRH and KISS1 receptor (GPR54) (Figure 3, panel c). Moreover, the expression of dynorphin (PDYN) and tachykinin 3 (TAC3) genes—negative and positive regulators of GnRH signaling, respectively—was not affected by HFD. The opioid receptor δ 1 (OPRD1)—one of the receptors for dynorphin—tended to increase in HFD hypothalamus (*p* = 0.06 vs. RD; Figure 3, panel c). Interestingly, TTh was able to restore KISS1 mRNA to RD levels, reduce the negative regulator PDYN and its receptor OPRD1, and increase the positive regulator TAC3 (Figure 3, panel c). Moreover, a positive correlation was observed between the mRNA expression of GnRH and of the following genes: AR (*r* = 0.391, *p* = 0.004), ERα (*r* = 0.479, *p* < 0.001), KISS1 (*r* = 0.363, *p* = 0.012), TAC3 (*r* = 0.417, *p* = 0.002), while a negative correlation was present with OPRD1 (*r* = −0.273, *p* = 0.052).

### 2.4. Modulation of Markers of Neurogenic/Neurodifferentiation and of the Melanocortin System Induced by HFD and TTh

**Figure 4:**
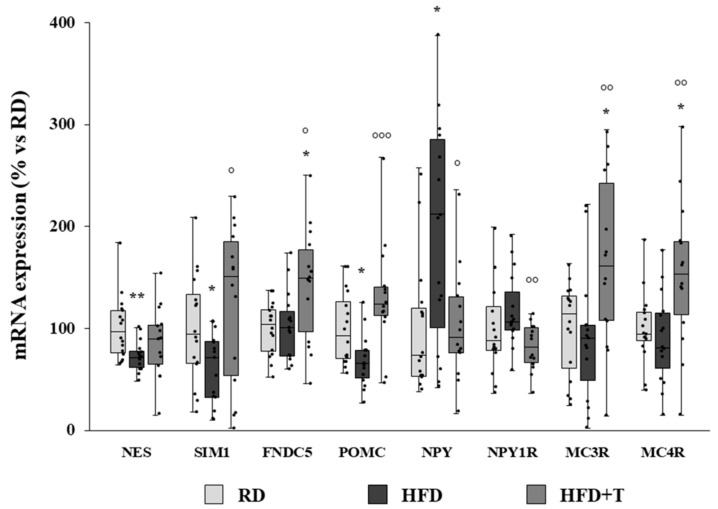
mRNA expression of neurogenic/neurodifferentiation markers and melacortin system-related genes in RD, HFD, and HFD + T rabbit hypothalamic samples, as evaluated by qRT-PCR. Data were calculated using the 2^−ΔΔCt^ comparative method, with the 18S ribosomal RNA subunit used as housekeeping gene for normalization, and they are reported as a percentage vs. RD as median ± interquartile range (*n* = 16 for RD, *n* = 15 for HFD, *n* = 14 for HFD + T). Statistical analysis between groups was performed with Kruskal–Wallis and post hoc Mann–Whitney nonparametric tests (* *p* < 0.05, ** *p* < 0.01 vs. RD; ° *p* < 0.05, °° *p* < 0.01, °°° *p* < 0.001 vs. HFD).

The qRT-PCR analysis of hypothalamic samples showed an HFD-induced reduction in the mRNA expression of nestin (NES), a marker of neural progenitor cells in the adult brain [32], and of the single-minded family basic helix–loop–helix transcription factor 1 (SIM1), a transcription factor required for the correct differentiation of the paraventricular, supraoptic, and anterior periventricular nuclei of the hypothalamus [33] (Figure 4). Although TTh did not significantly affect NES expression, it completely normalized SIM1 expression up to RD levels (Figure 4). Moreover, TTh strongly enhanced the expression of the FNDC5 gene—which encodes irisin, a protein exerting neurotrophic effects [34]—also up to a higher extent as compared to RD (Figure 4).

As shown in Figure 4, HFD altered the hypothalamic expression of genes related to the melanocortin system, determining a significant increase in NPY and a significant reduction in POMC mRNA expression. TTh significantly reverted the HFD effect by normalizing NPY and POMC expression to RD levels (Figure 4). TTh also significantly decreased the expression of NPY1R, while it increased MC3R and MC4R, thus promoting anorexigenic signaling (Figure 4). Moreover, POMC, MC3R, and MC4R gene expression positively correlated with GnRH gene expression (*r* = 0.320, *p* = 0.023; *r* = 0.497, *p* < 0.001; *r* = 0.473, *p* < 0.001, respectively), while NPY1R showed a negative correlation (*r* = −0.310; *p* = 0.025).

**Figure 5:**
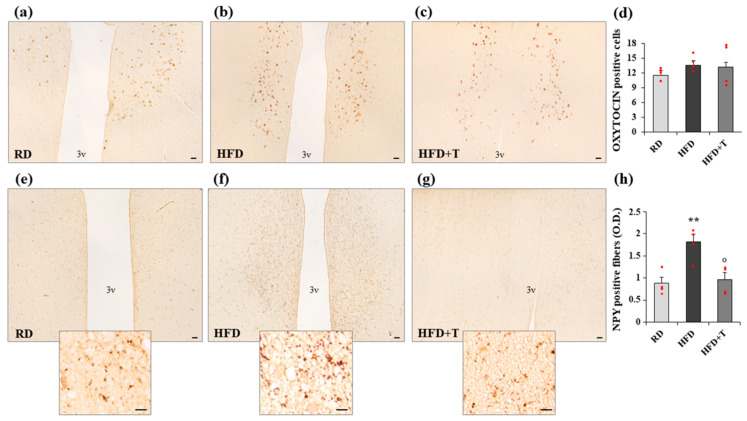
Immunostaining of oxytocin and neuropeptide Y (NPY) positivity in the hypothalamic paraventricular nucleus (PVN) from RD, HFD, and HFD + T rabbits. Representative images of coronal sections showing the presence of both oxytocin-positive neurons (**a**–**c**) and NPY-positive fibers (**e**–**g**, higher magnification in the inset) localized in the PVN, adjacent to the third ventricle (3v). (**d**) Quantification of oxytocin-positive neurons obtained by counting 10 fields in four different samples from each group in the PVN area (means ± SEM, *n* = 4 for each group); (**h**) Computer-assisted analysis of NPY-positive fibers, calculated in 10 fields of four different samples from each group (mean ± SEM, *n* = 4 for each group; ** *p* < 0.01 vs. RD; ° *p* < 0.05 vs. HFD). Scale bar = 50 μm (lower magnification) and 10 μm (higher magnification).

Immunohistochemical analyses of hypothalamic coronal sections confirmed the findings concerning NPY expression (Figure 5). We used a specific antibody against oxytocin in order to identify the specific area, adjacent to the third ventricle, corresponding to the paraventricular nucleus (PVN), where the main contingent of NPY fibers is directed to control energy homeostasis. The number of oxytocin-positive cells/field, counted in the PVN, was not affected by HFD and HFD + T treatments (Figure 5, panel d). Serial sections were then immunostained with NPY specific antibody, and the positive fibers were quantified with ImageJ software. As shown in Figure 5 (panels e–h), HFD significantly enhanced NPY-positive fibers in the PVN, while TTh restored the NPY immunopositivity to RD levels (Figure 5, panel h).

## 3. Discussion

In the present study, we confirm previous findings showing hypothalamic inflammation and alterations in an in vivo animal model of MetS [7,35], whose phenotype recapitulates the human one, including secondary hypogonadism, characterized by low plasma levels of both T and LH [26]. Moreover, we describe, for the first time, positive effects of TTh on the MetS-induced hypothalamic alterations, as already observed in other peripheral tissues [15,28,29,30,31].

HFD-associated hypothalamic inflammation has been demonstrated in both animal and human studies [3,4,5,6,7,8,9]. In HFD-fed animal models, several signaling pathways have been identified as candidate mediators of this alteration, including c-Jun N-terminal kinase (Jnk), nuclear factor-κB, Toll-like receptor 4 (TLR4), ceramide, and endoplasmic reticulum stress [36]. In fact, targeted hypothalamic disruption of these pathways decreased HFD-induced obesity, hypothalamic leptin resistance, and systemic insulin resistance [36], implying a relevant contribution of neuroinflammation. Disruption of the BBB permeability, occurring with HFD, is an important player in neuroinflammation, because it facilitates crossing of circulating inflammatory mediators, principally in the hypothalamic nuclei close to the third ventricle and median eminence [37].

In the present rabbit model of MetS, we previously demonstrated that TTh is able to ameliorate several MetS-induced peripheral derangements, by positively affecting metabolic parameters [26], including circulating TNF-α [15], and by decreasing the overall inflammatory status of several MetS target organs, such as the liver, prostate, and bladder [15,30,31]. In the present study, we confirm previous findings on a beneficial effect of T on glucose metabolism and insulin resistance, as well as on hypertension and on visceral adipose tissue accumulation [28,29,30,31]; however, above all, we here demonstrate, for the first time, its anti-inflammatory and positive effects also at the hypothalamic level.

Previous findings showed an increased expression of inflammatory markers such as COX-2, IL-6, and RAM11 and the activation of microglia in the hypothalamus of HFD-fed rabbits [7,35]. Herein, we demonstrated that TTh strongly counteracted HFD-induced inflammation and determined an overall amelioration of all the analyzed inflammatory parameters, with a normalization of the expression of IL-6 and IL-10 cytokines and of macrophage-related markers, along with a reduction in microglial activation.

These data are in agreement with some evidence that has shown a beneficial effect of sex steroids within the central nervous system (CNS) [38]. In particular, it has long been known that T, independently of the estradiol pathway, shows some neuroprotective properties [39], by increasing hippocampal neurogenesis and neuroprotection [40] and improving cognitive tasks and neuropathology in Alzheimer’s disease [41]. These effects are mediated by activation of the AR, which is widely expressed in the CNS, particularly in the hypothalamic regions where it is also responsible for the male HPG axis regulation [38].

Even if the neuroprotective effect of androgens was reported in a number of studies, only few documented an anti-inflammatory role in the CNS. For instance, Moser et al. demonstrated that TTh mitigated the HFD-induced microglial activation in middle-aged rats and astrocyte activation in aged rats [42]. More recently, Yang et al. showed that dihydrotestosterone (DHT) exerts anti-inflammatory effects in vitro and in vivo by inhibiting microglial activation and the release of proinflammatory factors, attenuating neuronal damage, and ameliorating cognitive impairment and motor dysfunction in lipopolysaccharide (LPS)-induced neuroinflammation in mice [43]. Interestingly, Atallah et al. demonstrated in male mice that chronic depletion of gonadal T leads to BBB dysfunction and to neuroinflammation. On the other hand, supplementation of T to castrated mice restored BBB integrity and almost completely abrogated the inflammatory features [44].

The inflammatory status of hypothalamus in MetS leads to some important functional alterations. In fact, we previously demonstrated that hypothalamic inflammation in HFD rabbits negatively affects the GnRH neuronal population and its main regulators [7,35]. In the present study, we found that TTh was able to counteract this phenomenon, acting on the circuits upstream to GnRH signaling and involving KISS1 neurons. It is known that a peculiar population of KISS1 neurons in the ARC, called KNDy, are regulated in an autocrine manner by the release of tachykinin 3 (also referred as neurokinin B) and dynorphin, which are stimulatory and inhibitory signals, respectively, for kisspeptin release and, consequently, for GnRH expression and secretion [45]. TTh restored the HFD-altered expression of KISS1, while it reduced the mRNA expression of dynorphin (PDYN) and its receptor (OPRD1), as well as enhanced tachykinin 3 (TAC3) mRNA, thus suggesting the restoration of KNDy signaling. Accordingly, GnRH mRNA expression correlated positively to KISS1 and TAC3 and negatively to OPRD1 mRNA levels.

As known, NPY/AgRP neurons and POMC/CART neurons, located in the hypothalamic ARC, regulate body weight and energy balance. Because of their proximity to the third ventricle and circumventricular organs, such as the median eminence, outside the BBB, they are more susceptible to peripheral metabolic and hormonal signals acting through delicate circuits that can be easily disrupted. Thus, in obesity and MetS, hypothalamic inflammation may cause resistance to the anorexigenic hormones leptin and insulin, leading to the defective regulation of food intake and energy expenditure [36]. Available data suggest that the initial events of hypothalamic inflammation induced by HFD could involve exactly an injury to neurons regulating energy balance circuits [36], particularly POMC neurons that dramatically decrease in number [4,23]. Moreover, HFD contributes to mitochondrial dysfunction in POMC neurons of male rodents decreasing the ability to process and secrete α-melanocortin (α-MSH) [46].

In line with these findings, we herein confirm an alteration of the melanocortin system in HFD-fed rabbits, with a reduction in anorexigenic POMC and an increase in orexigenic NPY [35]. In addition, a tight association between circulating T levels and the expression of POMC (positive correlation) and of NPY receptors (negative correlation) was previously detected [35]. We herein demonstrate that T determined a restoration of NPY and POMC expression, as well as a recovery of the expression of NPY1R. Moreover, MC3R and MC4R, mediating the anorexigenic signal, resulted upregulated by TTh.

The control of appetite and energy expenditure in the hypothalamus includes also a neuronal population located in the hypothalamic PVN and expressing SIM1 [47]. A sub-population of SIM1-positive neurons expresses MC4Rs and is, thus, responsive to α-MSH produced by POMC neurons of the ARC [47]. In rodents, loss of SIM1 neurons causes obesity with hyperphagia and decreased energy expenditure [48,49]. Moreover, SIM1 gene mutations also cause obesity and hyperphagia in both rodents and humans [50,51,52]. Lastly, SIM1 neurons in the PVN are damaged by an HFD in mice [53]. These observations indicate that SIM1 neurons have a central role in energy homeostasis and are in agreement with the reduction in SIM1 gene expression detected in the hypothalamus of HFD rabbits. Of note, TTh was able to counteract this negative effect of HFD.

Another interesting finding of this study was the effect of TTh in increasing the expression of FNDC5 gene, which encodes irisin, a recently discovered endocrine factor. Irisin is mainly secreted as a myokine and an adipokine, but it is also produced in the hypothalamus [54]. Knockdown of FNDC5 in neuronal precursors impairs development into mature neurons, suggesting a developmental role of FNDC5 in neurons [55]. Moreover, the colocalization of irisin and NPY in human hypothalamic sections of PVN was observed [56], along with its positive effect on POMC and CART expression in rats [57], thus suggesting a role in regulating the energy homeostasis at central level. Irisin is also a novel candidate factor for the regulation of reproductive function and puberty onset, with a stimulatory input on GnRH neurons [58]. Thus, the observed positive effects of TTh on FNDC5 gene expression could reflect a beneficial effect on both energy homeostasis and the reproductive axis.

On the other hand, it is well known that POMC and AgRP/NPY neurons also regulate GnRH neuron function [59] and, consequently, exert a crucial role in the signaling connecting nutrition/energetic status and reproduction. Hence, the positive effects of T noticed in both energetic metabolism and reproduction-related neuronal populations could reflect the amelioration of the whole circuit. Accordingly, in the present study, we also report that POMC, MC3R, and MC4R gene expression positively correlated whereas NPY1R negatively correlated with GnRH hypothalamic expression levels.

Our study might have clinical implication. In fact, the clinical opportunity to treat MetS-related hypogonadism with TTh, although supported by some [60], but not all [61], meta-analyses, has been recently questioned [62], suggesting that lifestyle modifications, more than TTh, should be the first line of intervention in this condition. Our previous preclinical study supports this view, because MetS-related hypogonadism can be fully reverted by aerobic physical exercise [35]. In fact, chronic, moderate physical activity restored discrete MetS-associated alterations at the hypothalamic and testicular levels, finally reverting the hypogonadal condition [35]. However, we also noticed that MetS rabbits were less able to perform physical exercise, because of an MetS-induced decreased muscle functional activity, which can be substantially counteracted by TTh [29]. Hence, an association between lifestyle modification and TTh seems to represent the ideal intervention in MetS-associated hypogonadism. The present study further supports this view, suggesting that TTh can preserve the hypothalamus from MetS-induced neuroinflammation, also protecting the complex machinery upstream to GnRH to be functionally operating when T-induced negative feedback is removed. In fact, present data suggest that T is able to preserve the upstream circuits that regulate GnRH, i.e., the KNDy neurons and the melanocortin system, from the HFD-induced insults. On the other hand, TTh, as expected, maintains a negative feedback on the HPG axis that results in suppressed LH levels. It is possible that a complete restoration of a normal HPG activity will be promoted once this negative feedback is removed by interrupting TTh. In addition, it is conceivable that the anti-inflammatory effect of T treatment could also have beneficial effects on hypothalamic neurons that control food intake and energy balance.

In conclusion, our data suggest that TTh in MetS-related hypogonadism has beneficial effects on inflammation at the hypothalamic level with additional positive outcomes on the hypothalamic circuits implicated in the control of energy metabolism and reproductive function. The identification of these effects adds new relevant insights into the comprehension of the complex and not fully elucidated mechanisms through which TTh improves several metabolic derangements characterizing MetS, as observed both in the present animal model [26,28] and in several clinical studies [63].

## 4. Materials and Methods

### 4.1. Animal Treatments

The HFD-induced MetS animal model was obtained as previously described [26]. Male New Zealand White rabbits (Charles River, Calco, Lecco, Italy), weighing ~3 kg, were individually caged under standard conditions in a temperature- and humidity-controlled room on a 12 h/12 h light/dark cycle. Water and food were unrestricted throughout the study. After 1 week, animals were randomly divided into two groups: (1) control rabbits continued to receive a regular diet (RD group; *n* = 20); (2) treatment rabbits received a high-fat diet for 12 weeks (RD implemented with 0.5% cholesterol and 4% peanut oil; HFD group; *n* = 40). A subset of HFD rabbits (*n* = 20) was supplemented with a pharmacological dose of T (30 mg/kg weekly i.m. for 12 weeks). Three animals (one of the HFD group and two of the HFD + T group) showed difficulties in diet adaptation and were excluded from the study. Blood samples were obtained via marginal ear vein in all groups at week 12 before euthanasia. The blood was immediately centrifuged at 1800× *g* for 20 min, and collected plasma was stored at −20 °C until assayed. OGTT was measured before sacrifice as previously described [26]. Mean arterial blood pressure (MAP) was measured using a polyethylene catheter inserted into a femoral artery at week 12, after sodium thiopental (trade name Pentothal sodium, 50 mg/kg) sedation. Afterward, the rabbits were euthanized with a lethal dose of sodium thiopental. Immediately after animal sacrifice, the brain was removed, and the hypothalamus was dissected and harvested appropriately for the subsequent analyses. In detail, hypothalamic samples for immunohistochemical analysis were collected from four animals for each group, immediately fixed in 10% buffered formalin and processed for paraffin embedding. The hypothalamic samples from the remaining animals were flash-frozen in liquid nitrogen and stored at −80 °C until use for RNA extraction and gene expression analysis. Prostate, seminal vesicles, and visceral adipose tissue (VAT) were collected from all animals, weighed, and stored at −80 °C.

Criteria to evaluate the prevalence of MetS were defined by an algorithm designed taking into account the presence, as a dummy variable, of one or more of the following factors: hyperglycemia, high triglycerides, high cholesterol, increased blood pressure, and visceral fat accumulation. Cutoffs for each factor were derived by the mean ± two standard deviations of the analyzed parameter, as measured in a large database of RD rabbits (*n* = 96); positivity for three or more factors identifies MetS [28]. Animal handling and total number of animals employed in the study complied with the Institutional Animal Care and Use Committee of the University of Florence, Italy, in accordance with Italian Ministerial Laws No. 116/1992 and No. 26/2014 (Protocol No. 123/2013-B date 21 May 2013 and Protocol No. 261/2019-PR date 29 March 2019), endorsing the principles of laboratory animal care. Animals were permanently monitored (on a 24 h basis) regarding their wellbeing, following the ARRIVE (Animal Research: Reporting of In Vivo Experiments) guidelines for reporting animal studies (www.nc3rs.org.uk/ARRIVE).

### 4.2. Measurement of Cholesterol, Triglycerides, Glycemia, T, and LH in Rabbit Serum

Plasma cholesterol, triglyceride, and glucose levels were measured using an automated system (ADVIA 2004 Siemens Chemistry System; Siemens Science Medical Solution Diagnostic, Tarrytown, NY, USA), as previously described [26]. Plasma T levels were measured by ECLIA (electrochemiluminescence immuno assay) using the Elecsys Testosterone II kit with an automated chemiluminescence system (Cobas 800; both Roche Diagnostic GmbH Mannheim, Germany) after appropriate extraction as previously described [35]. Plasma LH was measured using an ELISA kit according to the manufacturer’s instructions (Jérémy Decourtye, Repropharm Vet, Nouzilly, France).

### 4.3. Oral Glucose Tolerance Test

The OGTT was performed in accordance with the published method [26]. After an overnight fast, a 50% glucose solution was orally administered to the animals, at a dose of 1.5 g/kg. Blood samples were collected via the marginal ear vein before and 15, 30, and 120 min after glucose loading. Plasma glucose was measured as described above. The incremental area under the curve (iAUC) was calculated by using GraphPad Prism software v.5.0 for Windows (GraphPad Software, La Jolla, CA, USA).

### 4.4. RNA Extraction and Quantitative RT-PCR Analysis

Isolation of total RNA from rabbit tissues was performed using TRIzol reagent (Life Technologies, Paisley, UK) and a RNeasy Mini Kit (Qiagen, Hilden, Germany), both according to the manufacturers’ instructions. Complementary DNA (cDNA) synthesis was carried out using the iScriptTM cDNA Synthesis Kit (Bio-Rad Laboratories, Hercules, CA, USA) and quantitative real-time RT-PCR (qRT-PCR) amplification and detection were carried out using SsoAdvanced Universal SYBR^®®^ Supermix and the CFX96 Two-Color Real-Time PCR Detection System (both Bio-Rad Laboratories). Specific PCR primers for rabbit target genes were designed on sequences available at National Center for Biotechnology Information (NCBI) GenBank (https://www.ncbi.nlm.nih.gov/) or Ensemble Genome (http://www.ensembl.org). The 18S ribosomal RNA subunit was quantified with a predeveloped assay (Hs99999901_s1, Life Technologies) and used as the housekeeping gene for the relative quantitation of the target genes according to the comparative threshold cycle (Ct) 2^−ΔΔCt^ method [64], with some modifications. In detail, we used the untreated group (RD) as the calibrator in each analysis, so that by definition the calculations would provide the fold-change of the treated group relative to RD. Data are reported graphically as a percentage over the RD group, whose mean was set at 100% for direct comparison of each measurement.

### 4.5. Immunohistochemistry and Immunofluorescence

The paraffin-embedded hypothalamic blocks were sectioned in the coronal plane (5 µm). Deparaffinized and rehydrated sections were processed using standard immunohistochemical procedures, as previously described [65]. Briefly, sections were incubated overnight at 4 °C with the following primary antibodies: goat polyclonal COX-2 (1:100; Santa Cruz Biotechnology, Santa Cruz, CA, USA), mouse monoclonal anti-IL-6 (1:1000; Abcam Ltd., Cambridge, UK), mouse monoclonal anti-rabbit macrophage (RAM11, 1:80; Dako, Carpenteria, CA, USA), rabbit polyclonal anti-IBA1 (1:300, Wako Chemicals, Richmond, VA, USA), goat polyclonal anti-NPY (1:100; Santa Cruz Biotechnology), and rabbit polyclonal anti-oxytocin (1:800; Chemicon, Temecula, CA, USA). The slides were incubated with biotinylated anti-mouse (Thermo Scientific, Waltham, MA, USA), anti-polyvalent mouse, and rabbit (Thermo Scientific) or anti-goat (Vectastain Elite ABC Kit; Vector Laboratories Inc., Burlingame, CA, USA) secondary antibodies, followed by streptavidin–peroxidase complex (Thermo Scientific). The reaction product was developed with 3′,3′-diaminobenzidine tetrahydrochloride as chromogen (Sigma-Aldrich, Sant Louis, MO, USA). For IBA1 staining, a fluorescent labeled secondary antibody was used (Alexa Fluor 488 goat anti-rabbit, 1:200; Thermo Scientific). Negative controls were performed avoiding primary antibodies. Slides were evaluated and photographed using a Nikon Microphot-FXA microscope (Tokyo, Japan). The number of COX-2-, IL-6-, RAM11-, and oxytocin-positive cells was counted in 10 fields from four animals for each group. The quantification of NPY-positive fibers was performed in using ImageJ software (Fiji bundle, downloadable at https://imagej.net/) in 10 fields from four animals for each group. Results were obtained by calculating first the mean value for each animal within each group and then performing the statistical analysis on the basis of the mean value from four animals for each group.

### 4.6. Statistical Analysis

Statistical analysis was performed with a one-way ANOVA test (Kruskal–Wallis) followed by Mann–Whitney post hoc analysis or with an unpaired two-sided Student’s *t*-test, respectively, for non-normally and normally distributed parameters, to evaluate differences between groups. Results are expressed as the mean ± standard error of the mean (SEM) for normally distributed parameters and median with interquartile range for parameters with non-normal distributions. Correlations were assessed using Spearman’s methods. A *p*-value < 0.05 was considered significant in all the analyses. The establishment of MetS in the animals was evaluated as previously described [28]. Statistical analysis was performed with the Statistical Package for the Social Sciences (SPSS v. 26.0; SPSS Inc., Chicago, IL, USA).

Experimental procedures were carried out using the facilities of the Molecular Medicine Facility, Department of Biomedical Experimental and Clinical Sciences “Mario Serio” and Department of Experimental and Clinical Medicine, section of Human Anatomy, University of Florence.

## Figures and Tables

**Table 1:** Metabolic and hormonal parameters of experimental groups at week 12.

	RD (*n* = 20)	HFD (*n* = 19)	*P* HFD vs. RD	HFD + T (*n* = 18)	*P* HFD + T vs. RD	*P* HFD + T vs. HFD
**Total body weight (g)**	3890.5 (3753.5–3988.2)	3733.0 (3479.7–3861.2)	0.058	3788.0 (3506.5–4174.2)	0.558	0.548
**Blood glucose (g/L)**	0.95 (0.82–1.13)	1.82 (1.59–2.24)	**<0.0001**	1.49 (1.06–1.74)	**0.001**	**0.002**
**OGTT (iAUC)**	121.1 (98.8–157.8)	207.0 (187.2–221.9)	**<0.0001**	182.0 (170.2–229.0)	**0.001**	0.064
**Cholesterol (mg/dL)**	48.0 (29.0–49.0)	1464.0 (872.5–2,370.0)	**<0.0001**	1167.0 (769.5–1317.2)	**<0.0001**	0.095
**Triglycerides (mg/dL)**	65.0 (43.5–75.0)	168.0 (114.0–256.0)	**<0.0001**	137.0 (83.0–344.5)	**0.001**	0.504
**MAP (mmHg)**	85.0 (70.0–98.7)	150.0 (131.2–165.0)	**<0.0001**	113.1 (106.2–122.7)	**<0.0001**	**<0.0001**
**VAT** **(% of total weight)**	0.95 (0.83–1.10)	1.11 (0.93–1.22)	**0.011**	0.13 (0.07–0.25)	**<0.0001**	**<0.0001**
**T (nmoles/L)**	6.72 (4.42–11.10)	0.70 (0.69–1.19)	**<0.0001**	19.75 (9.68–39.40)	**0.001**	**<0.0001**
**LH (ng/mL)**	0.33 (0.12–0.65)	0.06 (0.05–0.14)	**0.004**	0.06 (0.04–0.10)	**<0.0001**	0.389
**Prostate weight** **(% of total weight)**	0.016 (0.012–0.021)	0.009 (0.007–0.013)	**0.001**	0.027 (0.021–0.029)	**<0.0001**	**<0.0001**
**Seminal vesicle weight (% of total weight)**	0.014 (0.011–0.021)	0.013 (0.011–0.016)	0.595	0.055 (0.043–0.064)	**<0.0001**	**<0.0001**

Results are reported as medians with quartiles in brackets. Bold text for *p* value indicates a statistically significant difference. RD = regular diet; HFD = high-fat diet; OGTT = oral glucose tolerance test; iAUC = incremental area under the curve of OGTT; MAP = mean arterial pressure; VAT = visceral adipose tissue; T = testosterone; LH = luteinizing hormone.

## Data Availability

Not applicable.

## Abbreviations

AR	Androgen receptor
BBB	Blood–brain barrier
CD68	Cluster of Differentiation 68
ER	Estrogen receptor
GLUT	Glucose transporter
GnRH	Gonadotropin-releasing hormone
GPR30	G protein-coupled Receptor 30
HFD	High-fat diet
HPG	Hypothalamic–pituitary–gonadal
IBA1	Ionized calcium binding adaptor molecule 1
IL	Interleukin
IRS-1	Insulin receptor substrate 1
MC3R	Melanocortin receptor 3
MC4R	Melanocortin receptor 4
MetS	Metabolic syndrome
PVN	Paraventricular nucleus
T	Testosterone
TNF-α	Tumor necrosis factor α
TTh	Testosterone treatment
